# Every Bit Hurts: Quantifiable Effects of Low‐Level Anthropogenic Disturbance on Movement, Habitat Selection, and Energetics of Blanding's Turtles (*Emydoidea blandingii*)

**DOI:** 10.1002/ece3.72536

**Published:** 2025-11-17

**Authors:** Reta Lingrui Meng, Keith Nahwegahbow, Patricia Chow‐Fraser

**Affiliations:** ^1^ Department of Biology McMaster University Hamilton Ontario Canada; ^2^ Lands and Resources Department Whitefish River First Nation Birch Island Canada

**Keywords:** anthropogenic disturbances, Blanding's turtle, energetic consequences, habitat selection, habitat use

## Abstract

Anthropogenic development negatively affects biodiversity worldwide, particularly wildlife with low fecundity, long lifespans, and extensive habitat requirements such as freshwater turtles. While large‐scale habitat degradation's effects on freshwater turtles are well‐documented, the impact of low‐level disturbances remains understudied, even though these subtler disturbances may alter movement patterns, increase energetic demands, and reduce reproductive success, threatening population viability. Understanding the impacts of all disturbance levels, including those considered minimal, is critical for effective conservation of sensitive species. In this study, we examined the response of the federally endangered Blanding's Turtle ( *Emydoidea blandingii* ) to a narrow range of low‐level human disturbances in Northern Mnidoo Gamii (Georgian Bay), Ontario, Canada that included a reference site with no visible disturbance (REF), a site experiencing moderately low disturbances (DIS1; with roads), and a site experiencing higher levels of anthropogenic disturbances (DIS2; roads, industrial development). Using radio telemetry, we tracked 14 individuals (501 relocations) in REF during 2021 and 2022, as well as seven individuals (199 relocations) in DIS1 and 13 individuals (367 relocations) in DIS2 during 2023 and 2024. Turtles in DIS2 exhibited significantly larger home‐range size, longer home‐range length, and greater daily distance traveled than those in REF. Significant habitat selection was observed only in DIS2 at the landscape scale, whereas turtles in DIS1 and REF showed no significant selection at either the landscape or home‐range scale. The increased movement in DIS2 was estimated to cost females the energetic equivalent of producing 1.85 more eggs per active season (18.5% of a full clutch). These findings highlight that even moderate habitat disturbances can impose substantial energetic burdens on freshwater turtles, and that there may be a disturbance threshold above which the long‐term population viability is compromised. Conservation strategies should prioritize minimizing even low levels of habitat degradation to support the viability of at‐risk freshwater turtle populations.

## Graphical abstract

This study found that moderately low human disturbances, such as roads and nearby development, increased movement and energy expenditure in endangered Blanding's Turtles, potentially reducing their reproductive output. These findings suggest that even low‐level habitat disturbances may threaten population viability. Conservation efforts should focus on minimizing habitat degradation to protect sensitive freshwater turtle populations.

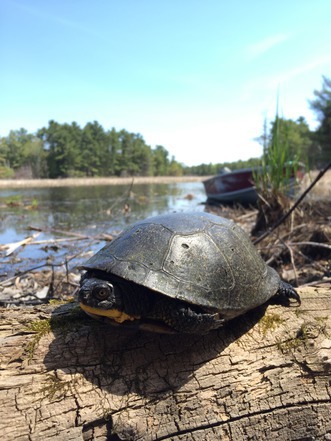

## 1 Introduction

Global biodiversity has declined by 73% from 1970 to 2020, with no indications of this trend slowing down (World Wildlife Fund 2024). Simultaneously, anthropogenic stressors on ecosystems and species at risk continue to intensify, exacerbating biodiversity loss (Butchart et al. 2010). Among these stressors, habitat loss and fragmentation remain primary drivers of species declines (Jaureguiberry et al. 2022). Human activities such as wetland drainage, dredging, land conversion, and proliferation of transportation infrastructure greatly contribute to habitat degradation and disruption of habitat connectivity (Prakash and Verma 2022). These alterations disproportionately affect slow‐moving, low‐fecundity species of turtles with strong site fidelity, such as the eight freshwater species found in Canada, all of which are classified as federally or provincially at risk (COSEWIC 2016; Ministry of the Environment, Conservation and Parks 2019).

**FIGURE 1:**
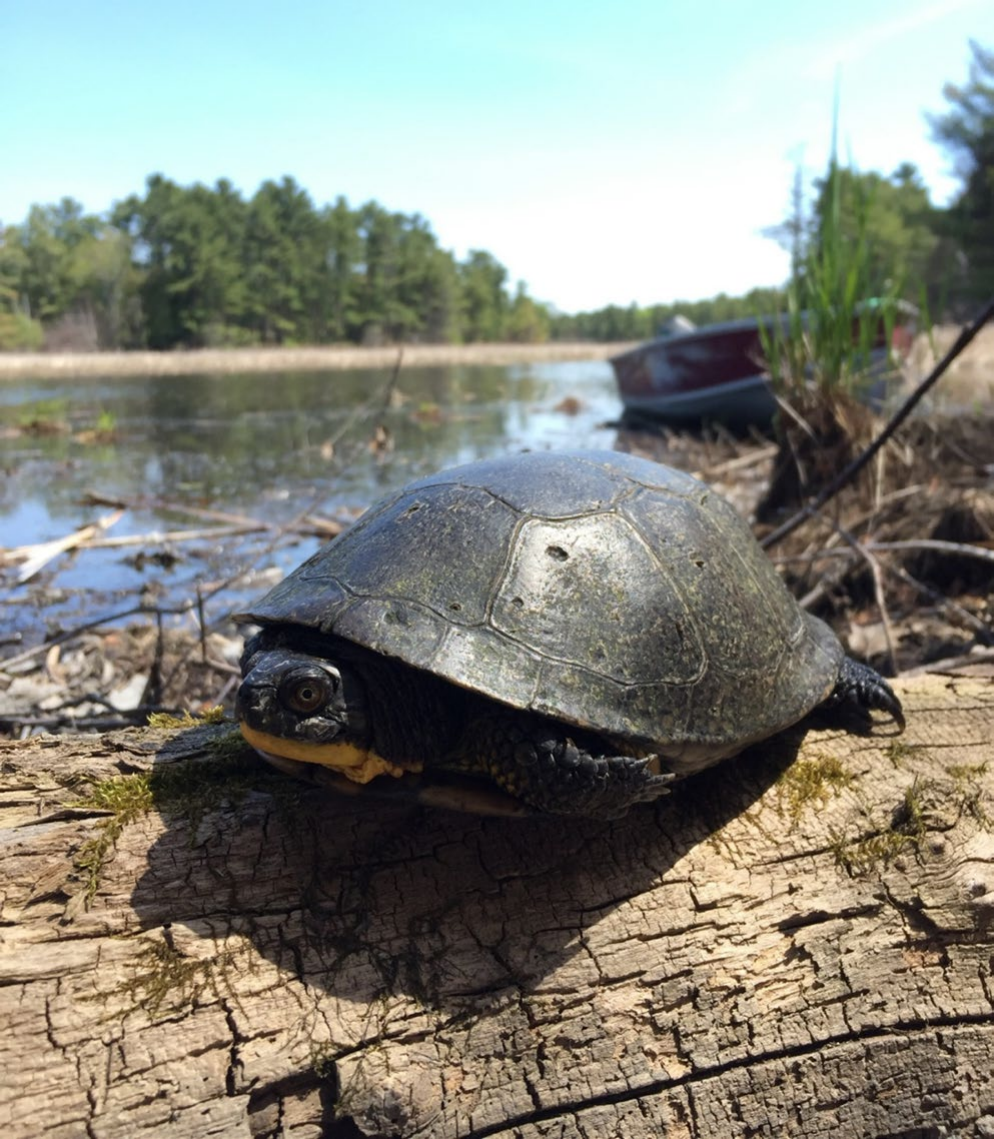
Blanding's Turtle ( *Emydoidea blandingii* ) basking on a log at the REF study site.

The Blanding's Turtle ( *Emydoidea blandingii* , BLTU; Figure 1) is a semi‐aquatic freshwater species primarily distributed across the Great Lakes–St. Lawrence region in Canada and the United States, with disjunct populations located in Nova Scotia, New York, Massachusetts, and Maine (COSEWIC 2016). BLTU rely on a diverse range of both terrestrial environments (e.g., forests, rock barrens, and ephemeral pools) and aquatic ecosystems (e.g., marshes, peatlands including bogs and fens, and swamps) throughout their range (COSEWIC 2016), occasionally also using deep open water habitats (Lehman 2023; Meng and Chow‐Fraser 2023). BLTU move regularly among wetlands and a variety of habitat types to access essential resources and complete all stages of their life cycle, including nesting, foraging, mating, and overwintering (Ministry of Environment, Conservation and Parks 2013). Throughout their range, habitat loss and road mortality are the leading causes of BLTU population declines (COSEWIC 2016). In southern Ontario alone, over 68% of wetlands have been lost since European colonization in the 1800s, primarily due to agricultural and urban expansion (Penfound and Vaz 2022). Habitat fragmentation and conversion to anthropogenic landscapes can reduce population viability through decreased nesting success (Mui et al. 2016), increased road mortality (Roberts et al. 2023), and diminished habitat suitability for BLTU life cycle requirements (Millar and Blouin‐Demers 2012).

A meta‐analysis of 11 studies on BLTU movement and habitat use across a broad disturbance gradient further revealed that as disturbance levels increased, BLTU exhibited changes in habitat selection, shifting from no significant selection in reference sites to significant selection across multiple habitat types in highly disturbed sites. These findings supported the hypothesis that in regions with sufficient resources and habitat availability, BLTU does not need to select for specific habitats to meet their life cycle requirements (Meng and Chow‐Fraser 2023). This pattern, however, contrasts with findings from O'Donnell and delBarco‐Trillo (2020), who conducted a meta‐analysis of 41 published studies and found that terrestrial vertebrates (primarily mammals and birds, with no freshwater turtles represented) generally exhibited reduced home‐range sizes in response to urban disturbance. They also reported no significant changes in home‐range sizes when disturbance levels remained minimal. The disparities between these two studies suggest that the relationship between disturbance and movement ecology may vary across taxonomic groups and highlight the need for further investigation into the mechanisms driving habitat use and movement patterns in freshwater turtles, particularly in the context of ongoing anthropogenic landscape changes.

Here, we build on Meng and Chow‐Fraser's (2023) hypothesis to propose a new paradigm where anthropogenic habitat disturbance not only affects habitat selection but also turtle movement and home range size. We hypothesize that in regions with abundant and diverse habitat types and minimal anthropogenic disturbances, BLTU have evolved to use the nearest, most accessible habitats, thus minimizing energetic costs. Conversely, as habitat fragmentation increases, we predict that BLTU will need to travel further to access suitable habitats, and this would result in greater movements and higher energetic expenditures as well as larger and longer home ranges. In essence, departures from reference conditions (i.e., no anthropogenic disturbances) should have a corresponding cost to the turtle that can be measured in terms of increased movement (and therefore energetic expenditures) and home range size that would likely contribute to statistically significant habitat selection by the individual or population.

It is challenging to use published data to test the above hypotheses since few studies provide data on habitat selection as well as home‐range size and movements. Additionally, available habitat classes vary across the species' geographic range (Meng and Chow‐Fraser 2023), making it difficult to conduct direct comparisons of home‐range size across studies. To remove the confounding effects of regional differences and to determine significant effects of low‐grade disturbances, we compared data for three adjacent populations living in close proximity (within 20,000 ha) in northern Mnidoo‐gamii (Georgian Bay), Ontario, Canada. One population lives in reference conditions without any roads or visible anthropogenic disturbances (REF), a second inhabits an area fragmented by roads but with no human development (DIS1), while a third lives in an area fragmented by roads, surrounded by industrial and urban development (DIS2). By choosing sites across this narrow range of low‐grade disturbances, we also want to determine if there is a disturbance threshold below which BLTU are no longer affected (Angoh et al. 2021; Fyson and Blouin‐Demers 2021; Markle and Chow‐Fraser 2018). This research has important implications for choosing priority areas for BLTU recovery and conservation. As well, since BLTU are considered an umbrella species in conservation science and practice (Herman et al. 2002), protecting their habitats also safeguards numerous other wetland‐dependent species, including other freshwater turtles, snakes, and amphibians.

## 2 Methods

This research is conducted as part of the Mshiikenh Ganawaabanjige (“Those Who Watch Over Turtles” in Anishinaabemowin) conservation program, a co‐created initiative between Whitefish River First Nation (WRFN) and McMaster University (MU) researchers. Our program aims to collaboratively identify and protect freshwater turtle populations, especially the BLTU (Meng et al. 2025, 2024). Whitefish River First Nation is an Anishinabek Ojibwe Nation and is located along the northern shoreline of Mnidoo‐gamii, where the community actively exercises its traditional stewardship rights. WRFN community members emphasize reciprocity and hold a deep connection with animals, water, and the land. The Mshiikenh Ganawaabanjige program is a community‐driven initiative that focuses on understanding, stewarding, maintaining, and, where possible, enhancing wetland and turtle population health within WRFN's traditional territory. Accordingly, all aspects of data collection, analysis, and dissemination were conducted collaboratively between the WRFN Lands and Resources department and MU researchers.

### 2.1 Study Site

Mnidoo‐gamii, the northeastern arm of Lake Huron, is an area rich in biodiversity and home to extensive coastal and upland wetlands. The Lake Huron–Mnidoo‐gamii coastal wetland grouping accounts for nearly 30% of the total wetland area across all five Great Lakes (Chow‐Fraser 2008). While this region remains relatively healthy and supports large populations of species at risk, emerging threats from cottage development, fluctuating water levels, and increasing human population density jeopardize the long‐term viability of these species (Chow‐Fraser and Croft 2015; Leblanc et al. 2014; Midwood and Chow‐Fraser 2012; Montocchio and Chow‐Fraser 2025).

**FIGURE 2:**
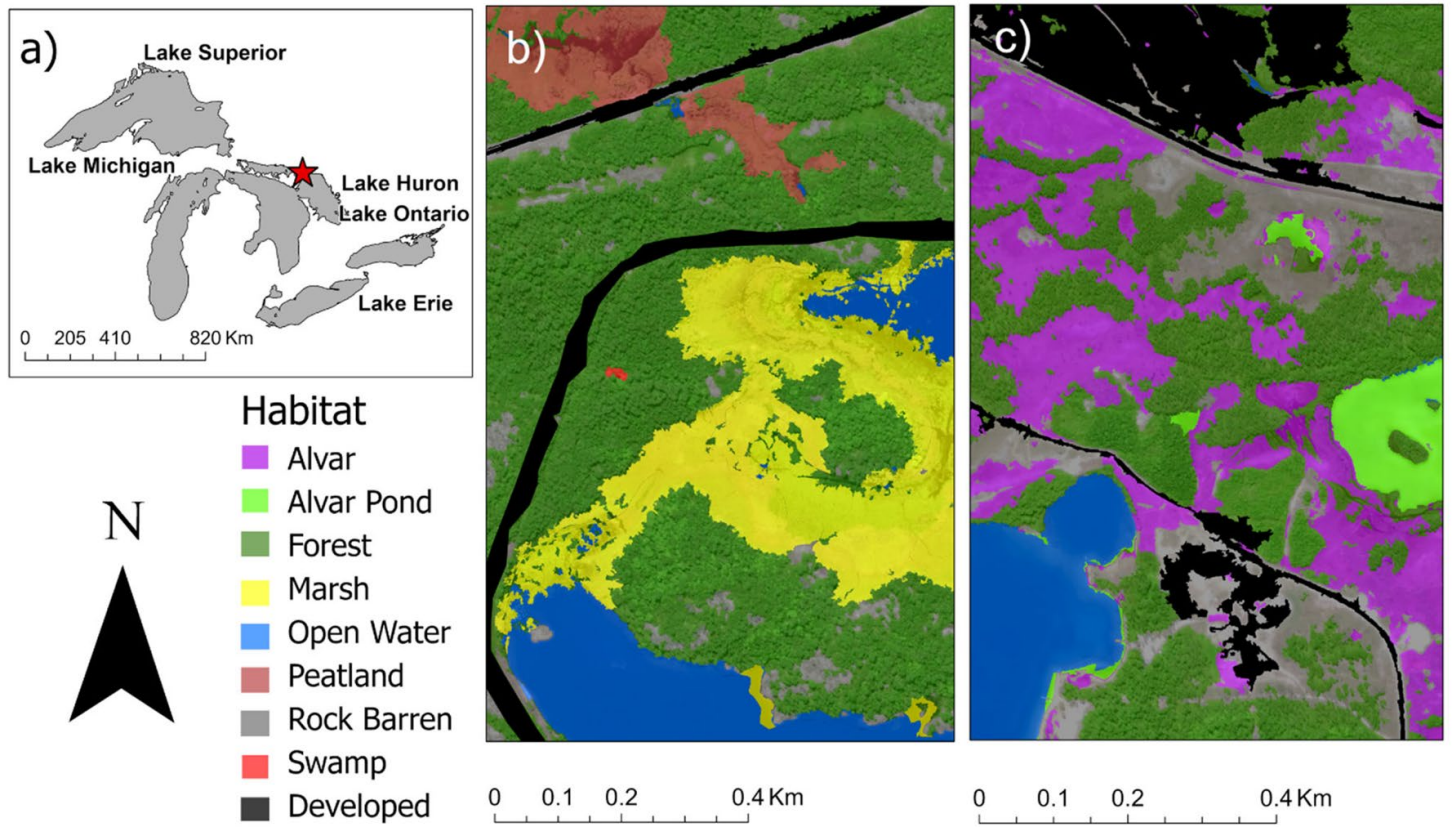
(a) Location of study site in the Mnidoo‐gamii (Georgian Bay, Ontario, Canada). (b) Habitat characteristics of the Blanding's Turtle found in DIS1, showing low to medium‐level disturbance with an adjacent railroad and highways (highlighted as Developed habitat in black). (c) Habitat characteristics of DIS2, showing highest level of disturbance with adjacent highways and land developed for industrial purposes (highlighted as Developed habitat in black).

The three sites in this study represent a gradient of human disturbance (Table 1, Figure 2). It is important to note that this disturbance gradient is only a narrow range within the large gradient of disturbance conditions documented for BLTU occurring in the Great Lakes–St. Lawrence region (see Meng and Chow‐Fraser 2023). For example, areas in southern Ontario and the northeastern United States experience much higher levels of human disturbance than does the most impacted site in this study. Study sites were selected through a collaborative process involving semi‐structured interviews with WRFN elders, land users, and discussion with WRFN Lands and Resources department staff. These steps allowed us to identify areas of high cultural significance and ecological value for turtles, ensuring that the research aligned with both community priorities and conservation objectives. Areas were prioritized based on their relevance to ongoing WRFN land use planning (e.g., potential development sites, contamination concerns) and their observed ecological value for turtles. Many of these sites were historically recognized by community members as important turtle habitats, with multiple generations witnessing high turtle activity in these regions. Detailed collaboration and methodological framework are available in Meng et al. (2025).

**TABLE 1:** Habitat disturbances and anthropogenic infrastructures identified across three Northern Mnidoo‐gamii (Georgian Bay) study sites with Blanding's Turtle ( *Emydoidea blandingii* ) populations, varying along a gradient of disturbance levels.

Site	Road length	Number of anthropogenic dwellings	Disturbance type	Disturbance class
Site 1–REF	0 km	~15 cottages	No visible disturbances	None
Site 2–DIS1	1.5 km highway 2.5 km railway track	~20 cottages + houses	Roads	Residential and Urban
Site 3–DIS2	6.5 km highway 9.5 km local roadway	~50 houses + 1 small‐scale industrial plant	Industrial/Urban Suburban/Residential Roads	Residential and Urban

The Reference (REF) site is located within an isolated archipelago along the northern shoreline of Mnidoo‐gamii (Meng and Chow‐Fraser 2023; Table 2). Accessible only by boat, this site experiences minimal anthropogenic disturbance, primarily limited to small pockets of shoreline cottage development. The region encompasses approximately 500 islands, with the study area situated on one main island featuring diverse habitats, including coastal cattail marshes, inland lakes, peatlands, rock barrens, upland forests, and open water. There is no large‐scale habitat fragmentation or degradation at this site, and the availability of varied natural habitats provides refuge for BLTU populations. A complete site description is available in Meng and Chow‐Fraser (2023).

**TABLE 2:** Land cover proportions and area across three Northern Mnidoo‐gamii (Georgian Bay) study sites with Blanding's Turtle ( *Emydoidea blandingii* ) populations, with varying levels of anthropogenic disturbance.

Land‐cover type	REF		DIS1		DIS2	
	ha	%	ha	%	ha	%
Alvar	19.75	9.17	0.00	0	0.00	0
Alvar pond	13.85	6.43	0.00	0	0.00	0
Forest	100.37	46.61	83.80	35.30	89.13	35.07
Marsh	8.70	4.04	78.38	33.02	58.63	23.07
Open water	36.31	16.86	60.58	25.52	87.91	34.59
Peatland	1.33	0.62	4.84	2.04	12.17	4.79
Rock barren	16.86	7.83	8.81	3.71	6.30	2.48
Swamp	5.28	2.45	0.59	0.25	0.00	0
Developed	12.89	5.99	0.38	0.16	0.00	0
Total area	215.34	100.00	237.38	100.00	254.16	100.00

The DIS1 site is characterized by cattail coastal marshes, open water, rock barrens, and upland forests (Table 2). A decommissioned railway track bed, currently undergoing decommissioning and remediation, is situated within this site, adjacent to a main cattail marsh. Various turtle species (including BLTU) use this linear feature for nesting and movement, which can increase predation risk and vehicular collision rates, as the track bed is intermittently used as a recreational ATV trail and serves as a travel corridor for predators including coyotes, black bears, raccoons, and red foxes (Meng, Nahwegahbow, and Chow‐Fraser, unpublished data). A 1.5 km stretch of provincial highway also exists within this site, and radio‐tracked turtles have crossed this highway above and underneath via culverts.

The DIS2 site is dominated by upland forests, alvar ecosystems, alvar clay ponds, fragmented forest patches, small pockets of coastal cattail marshes, and some human development, including an industrial processing plant, which is associated with 2.5 km of unpaved roads, several buildings, and truck traffic (approximately 5 trucks/h at peak times; Table 2). The region supports 50 year‐round residences and is bisected by approximately 6.5 km segment of a two‐lane highway (maximum speed 80 km/h).

### 2.2 Turtle Capture, Tagging, and Radio Telemetry

Data for 14 BLTU caught in REF (May and June of 2021 and 2022) are taken from Meng and Chow‐Fraser (2023). Data from 7 turtles in DIS1 and 13 turtles in DIS2 were obtained collaboratively by the WRFN Lands and Resources Team and the MU research team during May and June in 2023 and 2024. Asemaa (tobacco) was placed down alongside a prayer prior to beginning our work on the land each day, in accordance with WRFN research protocol under the guidance of community elders. We used baited hoop nets, visual surveys, and opportunistic hand capture at all three sites. In DIS1 and DIS2, we relied on place‐based knowledge from WRFN land users to guide us in identifying priority wetlands to survey based on their sightings of turtles in the past when spending time on the land (i.e., hunting, trapping, fishing, etc.). We used secondary morphological features including plastron concavity and position of cloacal opening to determine the sex of mature adults (Hamernick 2000; Innes et al. 2008) and recorded body mass and any visible injuries or deformities of all individuals captured. We also approximated the age of hatchlings and juveniles by counting growth annuli on plastral scutes when visible (Congdon et al. 1993). At this step, we introduced our good intentions and gratitude to each mshiikenh, in accordance with WRFN's culturally sensitive animal use protocol (Meng et al. 2025). Once weight is recorded to ensure the weight of the radio tag does not exceed more than 5% of an individual's body mass, we attached an AI‐2F radio transmitter (Holohil Systems Ltd., Carp, ON, Canada, 19 g) to the rear marginal scutes using WaterWeld Epoxy (J‐B Weld, Sulfur Springs, Texas, US) and superglue. Since the white epoxy could increase the visibility of the tag and elevate predation risk to the turtle, we used a black marker to camouflage the white epoxy. As a last step, we released each turtle back to where we originally found it within a few hours of capture.

Throughout the summers (between capture date and end of August), we located each turtle 2–3 times per week using a Lotek Biotracker Receiver and accompanying 3‐element Yagi antenna (Lotek Wireless, Newmarket, ON, Canada) (Table 3). We also collected additional relocations in October and February each year to confirm BLTU overwintering habitat. Although all turtles were tracked for the entire active season over 2 years, we had a few missing points when a transmitter either fell off or malfunctioned on two individuals. Geographic coordinates for each relocation were recorded with a handheld tablet equipped with an internal GPS device (Samsung Electronics, Suwon‐si, South Korea, precision 3–5 m). We collected 501 relocations for 14 individuals at REF across 2021 and 2022, 199 relocations for 7 individuals at DIS1 across 2023 and 2024, and 367 relocations for 13 individuals at DIS2 across 2023 and 2024. Data collected for the same turtle in different years were treated as independent samples to increase sample size (Edge et al. 2010).

**TABLE 3:** Tracking start and end day for radio telemetry of Blanding's turtles ( *Emydoidea blandingii* ) across three study sites in Northern Mnidoo‐gamii (Georgian Bay).

Site	Year	Start day	End day	Mean tracking duration (# of days)	Number of BLTU tracked
REF	2021	May 10th	October 1st	142 ± 1.48	F = 3, M = 3
	2022	May 10th	July 30th	140 ± 12.09	F = 7, M = 7
DIS1	2023	May 5th	October 15th	162 ± 2.51	F = 2, M = 2
	2024	May 8th	August 29th	113 ± 0.5	F = 4, M = 2
DIS2	2023	May 3rd	October 15th	162 ± 1.73	F = 3, M = 4
	2024	May 7th	August 28th	111 ± 7.07	F = 8, M = 3

### 2.3 Home Range

We calculated home range size using 100% Minimum Convex Polygons (MCP), a widely accepted method to estimate home range, which allows for comparison with other studies (Hamernick 2000; Markle and Chow‐Fraser 2014; Meng and Chow‐Fraser 2023). We also used ArcGIS Pro 2.0 (ESRI, Redlands, California) to calculate home range length, estimated as the maximum distance between relocation points (Jones 1996). We calculated home range size and length of each turtle for each year.

We first confirmed the normality of our data using a Shapiro–Wilk test (Home‐range size: *W* = 0.98, *p*‐value = 0.95; Home‐range length: *W* = 0.98; *p*‐value = 0.89), and then compared significant differences in home‐range size and home‐range length by site and sex among the three study sites using a two‐way ANOVA in R (version 4.1.2., R Core Team). Turtle 115 made an unusually long foray in May 2024, traveling 8 km away from the location of its initial sighting, which greatly increased its home range to 4.5 times larger than the mean size and 2.5 times longer than the mean length of male turtles found within the DIS2 site. When significance was detected, we conducted a post hoc Tukey test to determine the significance of differences between the mean of all possible group pairings.

### 2.4 Movement Metric

We used the as.ltraj function from the adehabitatLT package in R (version 4.1.2., R Core Team) to determine mean Daily Distance Traveled (DDT) as a metric for turtle movement across the active season. DDT was calculated for each individual as the mean of the distance between sequential locations divided by the number of days between those locations (Maddalena et al. 2020). We used 2021 and 2022 data for the REF site, and 2023 and 2024 data for the DIS1 and DIS2 sites and treated each year as independent samples to maximize our sample size. In the linear mixed effects model, site and sex were the fixed effects, DDT was a response variable, while the identity of turtles was included to control for individual variation in movement (Bolker et al. 2009; Maddalena et al. 2020). This helped us determine differences between sex and among sites for DDT. We performed post hoc pairwise comparisons using emmeans for factors that showed significant main effects in the linear mixed effects model, to determine which group means differed.

### 2.5 Habitat Classification

We used a habitat map for the REF site that had been created with high‐resolution satellite image data acquired between April 2nd—June 1st, 2021 (Central Ontario Orthophotography Project, 0.2 m resolution) and that yielded a 92.7% overall accuracy for five habitat classes that included rock barren formed from Canadian Shield granitic bedrock, deciduous‐coniferous mixed forests, cattail‐dominated coastal marshes, peatland wetlands, and open water (see Meng and Chow‐Fraser 2023). For DIS1 and DIS2, we created similar habitat maps using high‐resolution satellite image data (0.5 m resolution Worldview‐2 acquired in 2021) and geographic object‐based image analysis (GEOBIA) through an open‐source combination of FOSS4G package Orfeo ToolBox (French Centre National d'Etudes Spatiales, Paris, France) and QGIS software. We completed layer stacking using the multispectral Worldview‐2 Imagery and the Ontario Digital Elevation Model (DEM; 2 m resolution) acquired on September 1st, 2021, and generated a normalized difference vegetation index (NDVI) layer to create a 6‐layer composite image (De Luca et al. 2019). We then completed a LargeScaleMeanShift segmentation to segment the image into vector objects for classification and used a Support Vector Machine algorithm to produce a classification map with nine habitat types: Forest, rock barren, marsh, swamp, peatland, open water, alvar pond, alvar, and developed areas. To decrease confusion between rock barren and developed areas due to similar spectral profiles, we manually delineated the developed areas and masked this out prior to analyses for the DIS1 and DIS2 study sites. For image classification and accuracy assessment, we collected ground‐truth data during both 2023 and 2024 field seasons at DIS1 and DIS2. Although ground‐reference points were collected several years after the image had been acquired, we ensured that the ground‐truth data were collected at the same time of year (leaf‐on conditions during mid to late August). We also gathered additional ground reference points from visual interpretations of high‐resolution satellite imagery conducted together between MU researchers and WRFN land users and elders who live in relationship with the land on a daily basis. The overall accuracy of our classification was > 90%, which allowed us to use the habitat classification map in our study for analysis.

### 2.6 Macrohabitat Selection

We analyzed habitat selection at the biologically relevant second‐order (landscape; selection of individual home ranges from population range) and third‐order (home range; selection of individual locations from individual home range) selection scale. It is important to investigate habitat selection at multiple scales because selection at one scale reveals unique information that is not revealed at other scales. For example, selection at the landscape scale reveals patterns in connectivity and population‐level trends that are biologically relevant while the site‐level scale reveals information that offers insight into decisions that an animal makes while foraging, mating, and nesting (Johnson 1980; Mayor et al. 2009).

We followed a similar method used by Angoh et al. (2021) and Meng and Chow‐Fraser (2023) to analyze habitat selection for all three sites. We first delineated the population home range by creating a 100% MCP around all relocations acquired for each site. We then determined individual home range following the method of Angoh et al. (2021) by creating simulated habitat kernels for each turtle. We determined individual location by placing a buffer around each turtle relocation equivalent to the size of the average DDT (representing average daily turtle movement) within each population. Second‐order selection was then used to examine the proportion of habitat types found in the population and individual home ranges, while third‐order selection was used to examine the proportion of habitat types found in the individual home range and around individual relocations (further details found in Angoh et al. 2021 and Meng and Chow‐Fraser 2023).

To determine if significant selection occurred, we used compositional analysis to create a ranking matrix and test for habitat selection (Aebischer et al. 1993), which is a method commonly used to determine habitat selection in freshwater turtles (Delay et al. 2023; Markle and Chow‐Fraser 2014; Rasmussen and Litzgus 2010). This is a two‐step process in which the overall statistical significance of selection is tested with a Wilks' Lambda, which informs us if the animal is selecting habitat in a non‐random fashion. Afterwards, a ranking matrix is built to provide pairwise comparisons of habitat use, showing the relative preference or avoidance of each habitat type (Aebischer et al. 1993). The matrix is interpreted such that triple positive (+++) signs indicate that the habitat type in the row is used significantly more than the habitat type in the column, while triple negative (−−−) signs indicate it is used significantly less. Note that although matrices can be produced for each analysis, only those associated with a significant Wilks' Lambda test contain meaningful information; that is, the pattern of habitat use is a significant departure from randomness (Calenge 2006).

## 3 Results

### 3.1 Home‐Range Size

**FIGURE 3:**
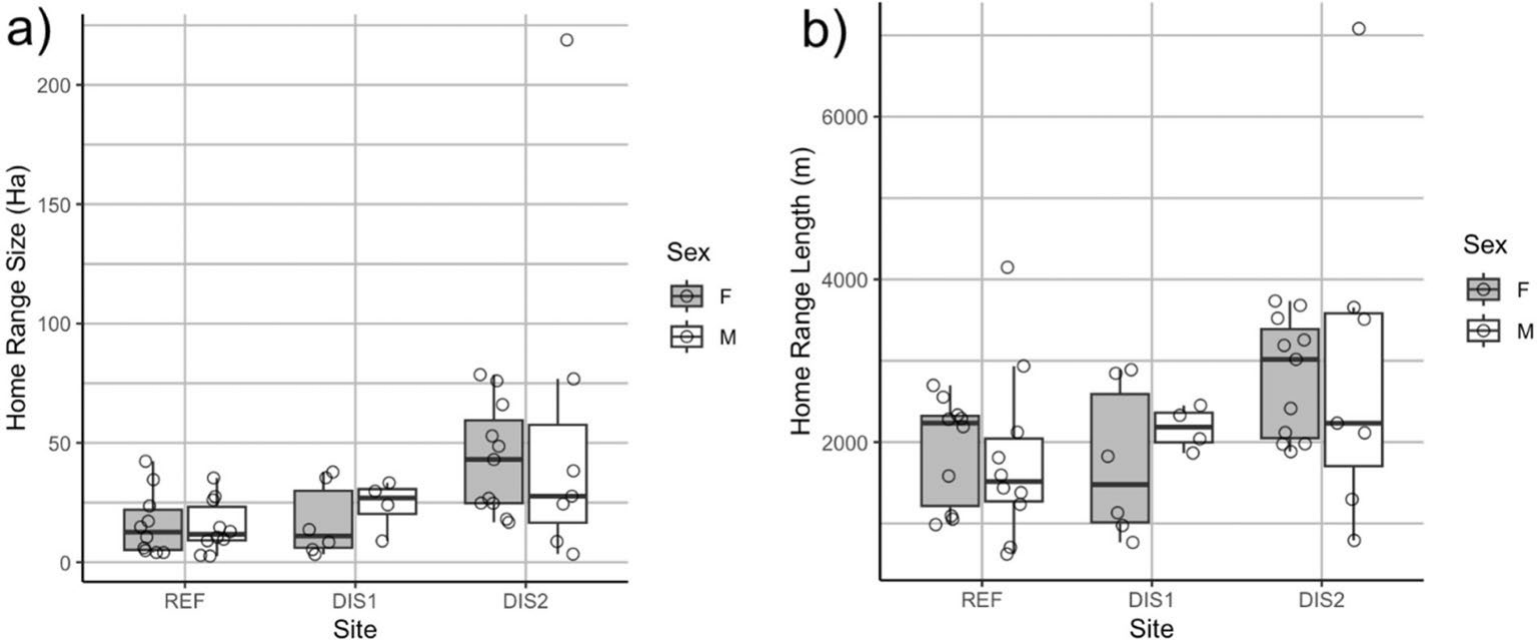
Box and whisker plots showing differences in (a) home‐range size and (b) home‐range length of male and female Blanding's Turtle across three study sites. Points overlaid on the boxes are the raw data. The thick line is the median, while the top and bottom of the boxes are the 75th and 25th percentiles, respectively. The ends of the whiskers are the maxima and minima, excluding outliers.

Controlling for site, home‐range size was not significantly different between sexes $\left(F_{1,42}=0.12,P=0.73\right)$; therefore, we pooled individuals from both sexes in our site comparison plots to increase our sample size. Home‐range size was significantly different among the three sites, and site explained most of the variability in home‐range size $\left(F_{2,42}=5.57,P<0.05\right)$. BLTU at the REF site had smaller home ranges (MCP, 15.66 ± 11.97 ha) compared to those at the DIS2 site (MCP, 48.58 ± 48.57 ha), while those in DIS1 (MCP, 19.98 ± 13.48 ha) did not significantly differ from either REF or DIS2 (Figure 3, Table 4). We reached the same conclusion whether or not Turtle 115 was included in the analysis. To assess whether our choice of home range estimator influenced results, we also calculated Kernel Density Estimators (KDE; Seaman and Powell 1996). The KDE analyses produced similar patterns, showing no significant differences in home range size between sexes $\left(F_{1,25}=0.06,P=0.81\right)$ but significant differences among sites $\left(F_{2,25}=4.64,P<0.05\right)$, consistent with the 100% MCP results.

**TABLE 4:** Home‐range size calculated using 100% MCP for Blanding's turtles ( *Emydoidea blandingii* ) in the three study sites from 2021 to 2024.

Sex	Site	Turtle ID	Size (ha)			
			2021^a^	2022^a^ ^s^	2023	2024
Female	REF	1	34.6	14.8		
	REF	3	23.6	6.0		
	REF	5	4.1	10.5		
	REF	10		42.3		
	REF	14		4.9		
	REF	16		17.2		
	REF	17		4.1		
	DIS1	102			3.3	8.4
	DIS1	109			5.3	35.4
	DIS1	117				13.7
	DIS1	118				37.8
	DIS2	105			24.8	16.7
	DIS2	106			48.6	18.0
	DIS2	110			78.6	76.0
	DIS2	112				24.7
	DIS2	114				26.8
	DIS2	119				43.0
	DIS2	120				52.9
	DIS2	121				66.0
Male	REF	2	12.9	35.3		
	REF	4	2.6	10.5		
	REF	6	9.6	26.1		
	REF	11		27.5		
	REF	12		14.6		
	REF	13		2.9		
	REF	15		9.0		
	DIS1	103			8.9	
	DIS1	108			24	29.8
	DIS1	116				33.3
	DIS2	100			38.3	
	DIS2	101			8.7	
	DIS2	104			27.7	76.8
	DIS2	111			24.4	3.4
	DIS2	115				218.8

^a^ Data previously reported in Meng and Chow‐Fraser (2023).

### 3.2 Home‐Range Length

Similar to home‐range size, we found no significant effect of sex on home‐range length when we controlled for site $\left(F_{1,42}=0.005,P=0.94\right)$; therefore, we also pooled individuals from both sexes in our site comparison plots to increase our sample size. Home‐range length was significantly different among the three sites $\left(F_{2,42}=4.59,P<0.05\right)$. BLTU at the REF site had significantly shorter mean home‐range length (1852.43 ± 862.82 m) than those at DIS2 (2858.44 ± 1371.71 m); however, BLTU in DIS1 had home‐range length (1912.37 ± 753.82 m) that did not differ significantly from either those in REF or DIS2 (Figure 3, Table 5). Again, including Turtle 115 in the analysis did not affect the test outcome.

**TABLE 5:** Home‐range length for Blanding's turtles ( *Emydoidea blandingii* ) measured at the three study sites from 2021 to 2024.

Sex	Site	Turtle ID	Length (m)			
			2021^a^	2022^a^	2023	2024
Female	REF	1	2698.0	2289.0		
	REF	3	2334.0	1093.0		
	REF	5	986.0	1583.0		
	REF	10		2553.1		
	REF	14		1052.4		
	REF	16		2189.4		
	REF	17		2281.0		
	DIS1	102			767.2	1131.8
	DIS1	109			975.7	2888.9
	DIS1	117				1825.8
	DIS1	118				2845.5
	DIS2	105			2413.5	1975.7
	DIS2	106			3254.9	1882.6
	DIS2	110			3680.7	3734.7
	DIS2	112				1980.4
	DIS2	114				2116.7
	DIS2	119				3188.4
	DIS2	120				3017.1
	DIS2	121				3522.3
Male	REF	2	1809.7	4148.0		
	REF	4	624.3	1379.7		
	REF	6	1436.9	2121.0		
	REF	11		2934.7		
	REF	12		1593.8		
	REF	13		707.0		
	REF	15		1235.3		
	DIS1	103			1866.2	
	DIS1	108			2040.5	2330.3
	DIS1	116				2451.8
	DIS2	100			3511.0	
	DIS2	101			1296.9	
	DIS2	104			2114.0	3654.6
	DIS2	111			2232.9	792.4
	DIS2	115				7083.2

^a^ Data previously reported in Meng and Chow‐Fraser (2023).

### 3.3 Daily Distance Traveled (DDT)

**FIGURE 4:**
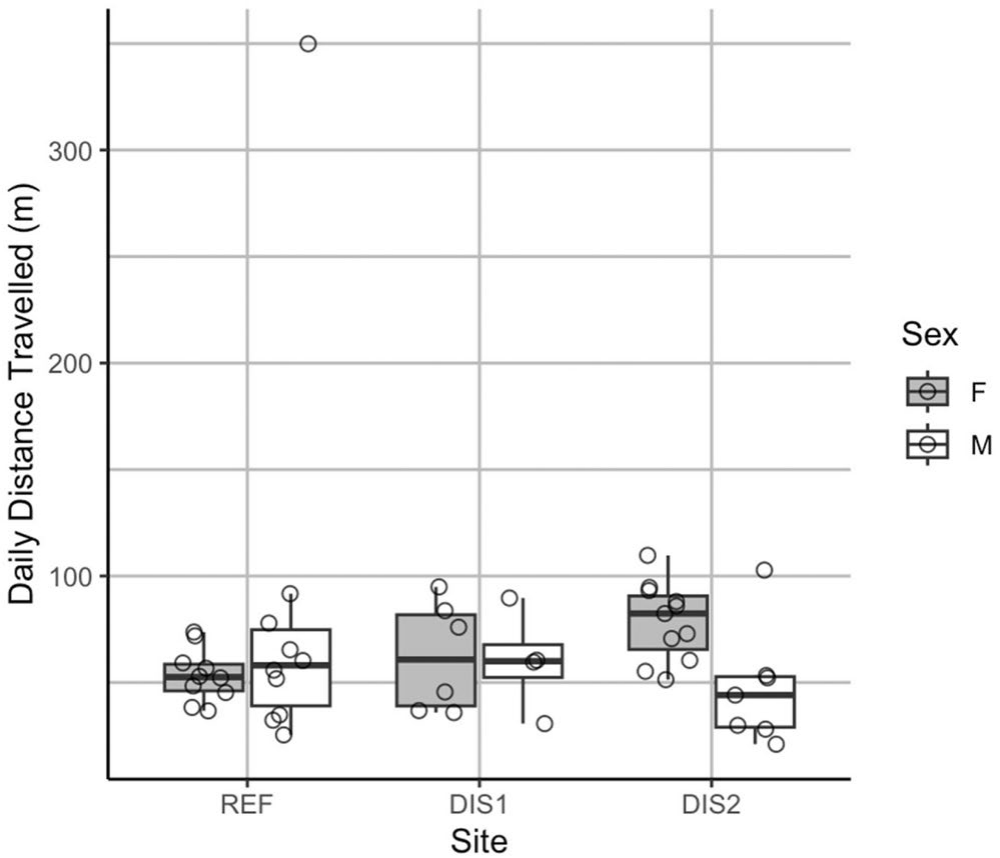
Box and whisker plots showing median (thick line) and the 75th and 25th percentiles (upper and lower lines of the box, respectively) of movement data (mean Daily Distance Traveled; DDT (m/d)) acquired between May and August for male (M) and female (F) Blanding's Turtle across the three study sites. The ends of the whiskers are the maxima and minima, excluding outliers.

Our linear mixed model showed that site had a significant effect on DDT, with turtles at the REF site traveling on average 120 m/day less than those at the DIS2 site $\left(\beta =-120.26,t=-3.341,P<0.05\right)$. There were, however, no significant differences in DDT between turtles at DIS1 and REF. To further explore pairwise differences, we used estimated marginal means (emmeans) for post hoc comparisons across sites and sexes. We found that females at DIS2 on average 120 m/day farther than females at the REF site ($\text{estimate}=120.26,\mathrm{SE}=36.1,t_{34.3}=3.33,P<0.5$; Figure 4, Table 6). This pattern remained consistent even when we added the unusually extensive movement of turtle 115 from the DIS2 site to the analysis.

**TABLE 6:** Daily distance traveled (DDT) calculated for Blanding's turtles ( *Emydoidea blandingii* ) at the three study sites from 2021 to 2024.

Sex	Site	Turtle ID	Mean (±SE) DDT (m/day)			
			2021^a^	2022^a^	2023	2024
Female	REF	1	56.7 ± 14.3	52.9 ± 17.8		
	REF	3	59.2 ± 23.3	48.4 ± 15.9		
	REF	5	36.7 ± 12.4	45.3 ± 14.1		
	REF	10		71.9 ± 27.0		
	REF	14		38.3 ± 9.5		
	REF	16		52.2 ± 8.5		
	REF	17		73.7 ± 39.7		
	DIS1	102			94.9 ± 207.8	36.8 ± 5.3
	DIS1	109			35.9 ± 41.1	45.6 ± 16.3
	DIS1	117				83.7 ± 23.6
	DIS1	118				76.0 ± 15.4
	DIS2	105			82.4 ± 153.1	88.0 ± 32.0
	DIS2	106			109.7 ± 192.4	93.2 ± 46.6
	DIS2	110			70.6 ± 92.7	94.6 ± 29.1
	DIS2	112				51.4 ± 12.2
	DIS2	114				60.4 ± 16.5
	DIS2	119				55.3 ± 17.8
	DIS2	120				73.0 ± 18.6
	DIS2	121				85.9 ± 17.5
Male	REF	2	55.8 ± 12.8	349.9 ± 254.0		
	REF	4	32.3 ± 5.6	51.8 ± 12.8		
	REF	6	60.3 ± 25.6	77.9 ± 20.0		
	REF	11		91.7 ± 31.3		
	REF	12		65.4 ± 19.5		
	REF	13		25.4 ± 6.4		
	REF	15		34.8 ± 7.3		
	DIS1	103			30.7 ± 39.7	
	DIS1	108			59.6 ± 48.1	89.7 ± 17.1
	DIS1	116				60.5 ± 13.4
	DIS2	100			44.1 ± 60.4	
	DIS2	101			29.9 ± 39.4	
	DIS2	104			52.2 ± 72.7	102.8 ± 22.4
	DIS2	111			28.1 ± 34.2	21.1 ± 6.4
	DIS2	115				53.3 ± 19.0

^a^ Data previously reported in Meng and Chow‐Fraser (2023).

### 3.4 Habitat Selection

Habitat selection patterns for BLTU varied across different sites and were influenced by the spatial scale of measurement. At the REF and DIS1 sites, BLTU did not exhibit significant habitat selection at either the landscape or home range scale (Table 7, Table 8). This indicates individual home ranges were established randomly within the population range, and that specific locations within individual home ranges were also selected randomly. By contrast, there was statistically significant habitat selection at the second‐order landscape scale for turtles living in DIS2 (λ=0.001,P<0.05; Table 8). The overall habitat preference rankings for BLTU in DIS2 at the second‐order landscape scale were as follows: alvar>forest > rock barren>alvar pond>developed > open water>peatland > swamp>marsh. At the landscape scale, BLTU predominantly selected for alvar, rock barren, and forest habitats. By contrast, we found no evidence that there was significant habitat selection at the third‐order home range scale at DIS2, (λ=0.196,P=0.194; Table 9).

**TABLE 7:** Results of a compositional analysis at the Reference Site (REF) at the (a) landscape scale, where available habitat is the minimum available area and used habitat is the turtle home ranges (Wilks lambda; *p* = 0.37) and (b) at the home range scale, where available habitat is the turtle home ranges and used habitat is the location of relocations (Wilks lambda; *p* = 0.734). “+” indicates that habitat type in the row is used less than habitat type in the column, and is not associated with statistical significance; “−” indicates that habitat type in the row is used more than the habitat type in the column, and is not associated with statistical significance; “0” indicates neutral selection; “+++” indicates that habitat type in the row is used significantly more than habitat type in the column, with a significant deviation from random (*p* < 0.05); and “−−−” indicates that habitat type in the row is used significantly less than habitat type in the column, with a significant deviation from random (*p* < 0.05).

(a)						
	Habitat type					
Habitat type	Marsh	Peatland	Open water	Rock barren	Forest	Rank
Marsh	0	+	+	+	+++	4
Peatland	−	0	+	+	+	3
Open water	−	−	0	+	+	2
Rock barren	−	−	−	0	+	1
Forest	−−−	−	−	−	0	0

(b)						
	Habitat type					
Habitat type	Forest	Peatland	Marsh	Open water	Rock barren	Rank
Forest	0	+	+	+	+	4
Peatland	−	0	+	+	+	3
Marsh	−	−	0	+	−	1
Open water	−	−	−	0	+	1
Rock barren	−	−	+	−	0	1

**TABLE 8:** Results of a compositional analysis for Disturbance Site (DIS1) at the (a) landscape scale, where available habitat is the minimum available area and used habitat is the turtle home ranges (Wilks lambda; *p* = 0.206) and (b) at the home range scale, where available habitat is the turtle home ranges and used habitat is the location of relocations (Wilks lambda; *p* = 0.704). See Table 7 for explanations of symbols in tables.

(a)								
	Habitat type							
Habitat type	Marsh	Open water	Forest	Developed	Rock barren	Swamp	Peatland	Rank
Marsh	0	+++	+++	+++	+++	+++	+++	6
Open water	−−−	0	+++	+++	+++	+++	+++	5
Forest	−−−	−−−	0	+	+	+++	+++	4
Developed	−−−	−−−	−	0	+	+++	+++	3
Rock barren	−−−	−−−	−	−	0	+	+	2
Swamp	−−−	−−−	−−−	−−−	−	0	+	1
Peatland	−−−	−−−	−−−	−−−	−	−	0	0

(b)								
	Habitat type							
Habitat type	Marsh	Open water	Developed	Swamp	Rock barren	Peatland	Forest	Rank
Alvar pond	0	+	+	+	+	+++	+	6
Developed	−	0	+	+	+	+	+++	5
Open water	−	−	0	+	+	+	+	4
Swamp	−	−	−	0	+	+++	+	3
Marsh	−	−	−	−	0	+	+	2
Peatland	−−−	−	−	−−−	−	0	+	1
Forest	−	−−−	−	−	−	−	0	0

**TABLE 9:** Results of compositional analysis for the Disturbed Site (DIS2) at the (a) landscape scale, where available habitat is the minimum available area and used habitat is the turtle home ranges (Wilks lambda; *p* < 0.05) and (b) at the home range scale, where available habitat is the turtle home ranges and used habitat is the location of relocations (Wilks lambda; *p* = 0.194). See Table 7 for explanations of symbols in tables.

(a)										
	Habitat type									
Habitat type	Alvar	Forest	Rock barren	Alvar pond	Developed	Open water	Peatland	Swamp	Marsh	Rank
Alvar	0	+	+++	+	+++	+++	+++	+++	+++	8
Forest	−	0	+	+	+	+++	+++	+++	+++	7
Rock barren	−−−	−	0	+	+	+++	+	+++	+++	6
Alvar pond	−	−	−	0	+	+	+	+	+	5
Developed	−−−	−	−	−	0	+	+	+	+	4
Open Water	−−−	−−−	−−−	−	−	0	+	+	+	3
Peatland	−−−	−−−	−	−	−	−	0	+	+	2
Swamp	−−−	−−−	−−−	−	−	−	−	0	+	1
Marsh	−−−	−−−	−−−	−	−	−	−	−	0	0

(b)										
	Habitat type									
Habitat type	Alvar pond	Developed	Open water	Swamp	Marsh	Peatland	Forest	Alvar	Rock barren	Rank
Alvar pond	0	+	+	+	+	+	+	+	+	8
Developed	−	0	+	+	+	+	+	+	+	7
Open water	−	−	0	+	+	+	+	+	+	6
Swamp	−	−	−	0	+	+++	+++	+	+	5
Marsh	−	−	−	−	0	+	+	+	+	4
Peatland	−	−	−	−−−	−	0	+	+	+	3
Forest	−	−	−	−−−	−	−	0	+	+	2
Alvar pond	−	−	−	−	−	−	−	0	+	1
Rock barren	−	−	−	−	−	−	−	−	0	0

## 4 Discussion

Habitat modification is a key contributor to reptile population declines globally (Doherty et al. 2020). Anthropogenic disturbances can impact freshwater turtles in various ways, including decreased occupancy probability (Fyson and Blouin‐Demers 2021; Paterson et al. 2021), altered behavior (Blanchett et al. 2024), and increased physiological stress (Selman et al. 2013). The direct impacts of anthropogenic disturbances on turtle home range and movement have been documented on the semi‐aquatic Eastern Box Turtles (*Terrapene carolina carolina*; Brown et al. 2021; Mancuso 2011) and the aquatic Softshell Turtles ( *Trionyx spiniferus* ; Plummer et al. 1997). This paper is the first to document the apparent relationship between home‐range size and length with disturbance level for the federally endangered semi‐aquatic BLTU (COSEWIC 2016). To facilitate a valid comparison across sites, we collected data at sites located in close proximity and conducted surveys within a 5‐year period. The fact that we found a significantly smaller home range for turtles in REF compared with DIS2, but no significant difference between REF and DIS1 may indicate there is a lower disturbance threshold, and this should be investigated further.

Turtle movements can be influenced by various factors, including seasonality (Bodie and Semlitsch 2000), sex (Aresco 2005), habitat connectivity (Becker et al. 2024), and temperature (Gordon 2023), but we do not think these factors confounded our results because all our data were collected at the same time each year, with equal representation of male and female turtles from the three sites. To further assess potential environmental variation among years, we gathered available historical weather data from the nearest Environment and Climate Change Canada weather station to our study area (Table 10) and conducted two‐way ANOVAs to determine whether precipitation and mean temperature differed significantly among years across the active season (May—August). We calculated total seasonal precipitation for 2021, 2022, and 2023 (371 mm, 252 mm, and 304 mm, respectively). Unfortunately, data for 2024 were unavailable at the time of analysis. Although these are relatively large interannual variations, we had insufficient data to assess the extent to which these differences may have contributed to significant differences in home range size. Nevertheless, we have assumed that these differences played a minor role since data from the REF site encompassed both a relatively wet year (2021) and a relatively dry year (2022), whereas data from the DIS1 and DIS2 sites represented intermediate precipitation conditions. Additionally, we found that mean monthly precipitation $\left(F_{1,8}=1.94,P=0.20\right)$ and mean monthly temperature $\left(F_{1,8}=0.29,P=0.60\right)$ did not differ significantly among years.

**TABLE 10:** Comparison of mean monthly temperature and total precipitation during the active season (May–August) from 2021 to 2023. All data were obtained from Environment and Climate Change Canada from the nearest weather station (~23.95 km from the study area; Massey Weather Station). Data for 2024 were not available at the time of acquisition.

Month	Year	Mean temperature (°C)	Total precipitation (mm)
May	2021	11.09	32.2
	2022	13.05	79.6
	2023	10.86	76.0
June	2021	18.23	121.0
	2022	16.54	41.2
	2023	17.42	46.2
July	2021	18.94	115.6
	2022	19.33	60.8
	2023	19.55	101.0
August	2021	20.17	157.0
	2022	18.80	70.2
	2023	17.39	81.6

The level of anthropogenic disturbance (particularly landscape changes) can significantly affect habitat connectivity and movements of turtles (Doherty et al. 2021; Hamilton et al. 2018). In this study, mean DDT for turtles at the most disturbed site (DIS2) was significantly higher than that at the REF. We followed the approach of Edge et al. (2010) and treated each individual (*n* = 14) in each year as independent observations to increase sample size. This approach is further justified because annual home ranges are year‐specific, with only a portion of individuals being tracked across multiple years. Additionally, females varied each year with respect to being gravid. Treating each turtle‐year combination independently allowed us to account for the reproductive status of the female. Therefore, the observed differences in movement patterns between REF and DIS2 are most likely attributable to differences in disturbance levels rather than to environmental or sampling biases.

We also found a relationship between habitat selection patterns and disturbance levels across sites. There was no significant departure from randomness with respect to habitat selection at either the landscape‐ or home‐range scale for turtles living in the REF and DIS1 sites, likely because they have abundant high‐quality resources (Meng and Chow‐Fraser 2023); by contrast, turtles in DIS 2 exhibited significant habitat selection at the landscape (second order) but not at the home‐range scale (third‐order). This suggests that turtles at the most disturbed site selected for landscape‐level habitat features to establish their home range, but did not choose specific habitat classes within their home range. We contrast this pattern with the highly selective behavior exhibited by turtles in highly disturbed areas (e.g., Southern Ontario), where there is significant habitat selection at both landscape and local scales due to habitat fragmentation and degradation (Markle and Chow‐Fraser 2018). These findings emphasize the importance of examining habitat selection at multiple scales to capture nuanced behavioral responses.

To explore the long‐term ecological consequences on BLTU populations living in the more disturbed habitat and contextualize our findings, we estimated the potential energetic costs incurred by female turtles having to move longer daily distances and traveling through larger home ranges. Since there were no energetic data specifically for BLTU, we used a conservative estimation method informed by Paterson et al. (2019) and Zani and Kram (2008). Specifically, we applied the mean cost of locomotion for ornate box turtles (8.0 J/kg·m; Zani and Kram 2008) to the average body mass of female BLTU in our study (1.4 kg), and this resulted in an estimated energy expenditure of 11.2 J/m. On the basis of our DDT data, female BLTU in DIS2 moved, on average, 120 m more per day than those in REF, an estimated additional energy expenditure of 1344 J per day. Over the course of the active season (May 1 to August 30; 121 days), this equates to 162.62 kJ of additional energy expenditure per turtle.

**FIGURE 5:**
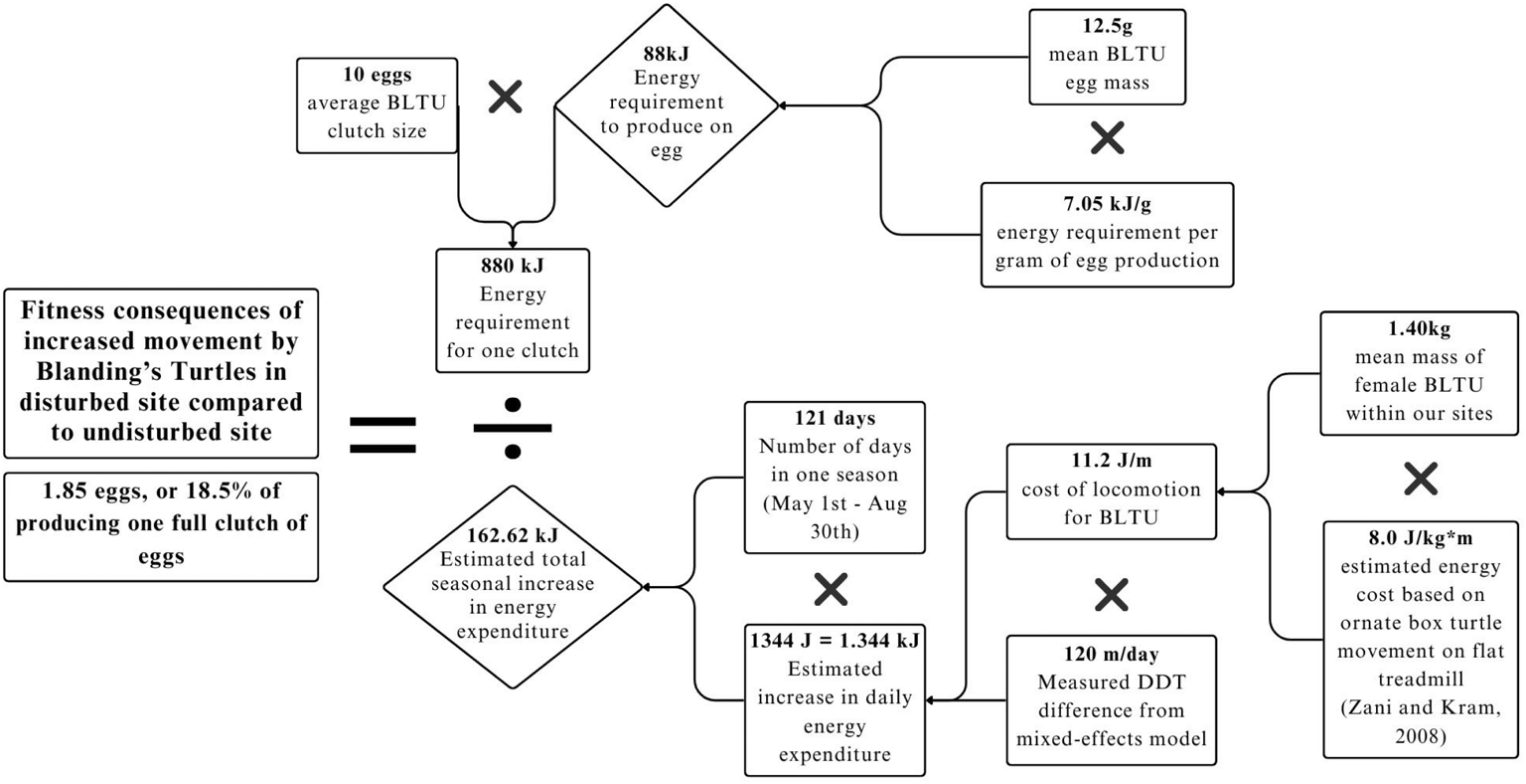
Flowchart outlining the steps and relevant equations used to calculate energetic expenses and to estimate the fitness consequences of increased movement in Blanding's Turtles.

To contextualize this cost, we can compare it to reproductive investment. Using a mean egg mass of 12.5 g and an energy content of 7.05 kJ/g (Congdon and Tinkle 1982; Paterson et al. 2019), the energy required to produce a single egg is approximately 88.125 kJ. The additional energy spent on increased movement in DIS2 is therefore equivalent to the energy required to produce 1.85 eggs, which represents 18.5% of the total investment needed for a full clutch of 10 eggs (Congdon and van Loben Sels 1993; MacCulloch and Weller 1988; Figure 5). This added energetic demand has meaningful implications for reproductive output and fitness. Female BLTU typically produce one clutch per year, and not all individuals nest successfully each season (Congdon et al. 2001; Ruane et al. 2008). Therefore, an energy deficit equal to a 20% reduction in fecundity would seriously erode annual reproductive success for that population. This conclusion is broadly supported by findings that increased energetic costs from environmental stressors often correlate with reduced fecundity and fitness (Shine 1995).

Our energetic estimates are likely underestimates of the real cost to BLTU traveling in DIS2. First, we used energy expenditures of ornate box turtles walking on treadmills, and this is less energetically demanding than animals negotiating natural landscapes that have uneven terrain, obstructive vegetation and/or inclement weather conditions. Secondly, our calculations only included travel during the active season, and did not include additional travel during the inactive season. Therefore, these should be considered conservative estimates, and represent the minimal energetic costs associated with turtles living in habitat with moderately low disturbance levels.

Meng and Chow‐Fraser (2023) first noted the relationship between habitat selection and the level of disturbances. Our study builds on this by demonstrating that even low‐level anthropogenic disturbances can affect BLTU populations, influencing home‐range size, movement patterns, and energetic expenditure, all of which have implications for population viability. Our findings highlight that BLTU have increased home‐range sizes and move longer distances in response to rising disturbance levels. This underscores the ecological significance of low to medium‐level disturbances, which have often been overlooked yet can substantially impact BLTU behavior and energetics. While our study confirms that low‐level disturbances can impact BLTU movement and energetics, further research is needed to identify if there is a disturbance threshold and if so, the precise level below which BLTU remain unaffected. Determining this threshold is critical for ensuring the long‐term population viability of BLTU populations, especially in regions where there is growing development pressure (e.g., in eastern and northern Georgian Bay). For sites experiencing low to medium‐level disturbances, restoration efforts remain feasible and should be prioritized to mitigate cumulative impacts. Overall, proactive measures to minimize habitat loss and fragmentation are essential for the long‐term viability of BLTU populations. Given that BLTU generally have large home ranges and diverse habitat needs that span multiple jurisdictions, it is crucial that all rightsholders and stakeholders work respectfully and engage actively in protecting BLTU populations for the next seven generations and beyond.

## Author Contributions

**Reta Lingrui Meng:** conceptualization (lead), data curation (lead), formal analysis (lead), funding acquisition (supporting), investigation (lead), methodology (lead), project administration (equal), visualization (lead), writing – original draft (lead), writing – review and editing (lead). **Keith Nahwegahbow:** conceptualization (supporting), data curation (supporting), formal analysis (supporting), funding acquisition (lead), investigation (supporting), project administration (equal), resources (supporting), supervision (equal), writing – review and editing (equal). **Patricia Chow‐Fraser:** conceptualization (supporting), data curation (supporting), funding acquisition (lead), project administration (equal), supervision (equal), visualization (supporting), writing – review and editing (equal).

## Conflicts of Interest

The authors declare no conflicts of interest.

## Data Availability

The data that support the findings of this study are openly available in The Open Science Framework at https://10.17605/OSF.IO/JTMQF.
